# Ursane-Type Triterpenes, Phenolics and Phenolic Derivatives from *Globimetula braunii* Leaf

**DOI:** 10.3390/molecules26216528

**Published:** 2021-10-28

**Authors:** Ayodeji Oluwabunmi Oriola, Adetunji Joseph Aladesanmi, Thomas Oyebode Idowu, Florence O. Akinwumi, Efere Martins Obuotor, Temilolu Idowu, Adebola Omowunmi Oyedeji

**Affiliations:** 1 Department of Chemical and Physical Sciences, Faculty of Natural Sciences, Walter Sisulu University, Mthatha 5099, South Africa; aoyedeji@wsu.ac.za; 2 Department of Pharmacognosy, Faculty of Pharmacy, Obafemi Awolowo University, Ile-Ife 220005, Nigeria; jaladesa@yahoo.com; 3 Department of Pharmaceutical Chemistry, Faculty of Pharmacy, Obafemi Awolowo University, Ile-Ife 220005, Nigeria; thomasidowu2010@yahoo.com; 4 Department of Pharmaceutics, Faculty of Pharmacy, Obafemi Awolowo University, Ile-Ife 220005, Nigeria; fakinwumi@oauife.edu.ng; 5 Department of Biochemistry and Molecular Biology, Obafemi Awolowo University, Ile-Ife 220005, Nigeria; efereo@yahoo.com; 6 Department of Chemistry, Parkers Building, University of Manitoba, Winnipeg, MB R3T 2N2, Canada; tidowu01@mail.ubc.ca

**Keywords:** *Globimetula braunii*, Loranthaceae, ursane-type triterpenes, phenolics, antioxidant, antimicrobial

## Abstract

*Globimetula braunii* is a hemi-parasitic plant used in African ethnomedicine for the management of microbial infections, rheumatic pain and tumors amongst others. We report the isolation and characterization of eight compounds with their antioxidant and antimicrobial activities. The air-dried powdered leaf was macerated in EtOH/H_2_0 (4:1). The extract was solvent-partitioned into *n*-hexane, EtOAc, *n*-BuOH and aqueous fractions. The fractions were screened for their antioxidant properties, using DPPH, FRAP, TAC and FIC assays. Antimicrobial analysis was performed using the micro-broth dilution method. The active EtOAc fraction was purified for its putative compounds on a repeated silica gel column chromatography monitored with TLC-bioautography. The isolated compounds were characterized using spectroscopic methods of UV, FT-IR, NMR and MS. Eight compounds (**1**–**8**) were isolated and characterized as 13,27-cycloursane (**1**), phyllanthone (**2**), globraunone (**3**), three phenolics: methyl 3,5-dihydroxy-4-methoxybenzoate (**4**), methyl 3-methyl-4-hydroxybenzoate (**5**) and guaiacol (**6**), as well as two phenol derivatives: 4-formaldehyde phenone (**7**) and 6-methoxy-*2H*-inden-5-ol (**8**). The study identified **4** and **6** as natural antioxidant compounds with potential as antimicrobial agents.

## 1. Introduction

*Globimetula braunii* (Engl.) Van Tiegh. (Family Loranthaceae) is a hemi-parasitic and epiphytic plant that derives water and mineral nutrients from its hosts by means of a specialized root system called “haustorium” [1]. It is commonly called “African Mistletoe” and locally called “Afomo Onisano” in Southwest Nigeria [2]. The plant is mostly found on dicot trees, such as *Piliostigma thonningii*, *Leucena leucocephala* and *Theobroma cacao,* where it becomes bushy and woody, growing up to 5 ft in diameter until the host tree withers [3]. At maturity, it produces reddish to reddish brown inflorescence with yellow patches in the form of a match sticks. It is widely distributed across tropical West African countries, such as Nigeria, Ghana, Benin Republic and Cameroun [4].

*G. braunii* is implicated in the African ethnomedicine for the management of microbial infections, wounds, cholera, hypertension, diabetes, rheumatism, ulcers and tumors [3,5,6]. Biological studies have shown the antioxidant, antimicrobial and cytotoxic potentials of the leaf extract and its ethyl acetate fraction [7,8,9]. The hepatic, hematologic, anti-lipidemic, oxytocic, anti-hyperglycemic and anti-cancer activities of the plant’s alcoholic, aqueous and ethyl acetate extracts have also been reported [5,6,10,11,12] with a paucity of information on its chemistry. Preliminary phytochemical reports showed that the plant contained phenolics, terpenes, flavonoids and sterols, while compounds, such as globrauneine A-F, lupeol, lupeol palmitate, β-sitosterol, friedelin, octanoic acid, lactones and flavonoids (quercetin, quercitrin, catechin, rutin and avicularin) have been reported in the plant [13,14].

We report, for the first time, the bioactivity-guided isolation and characterization of three ursane-type triterpenes, three phenolics and two phenol derivatives from the leaf of *G. braunii* along with their antioxidant and antimicrobial activities.

## 2. Results and Discussion

### 2.1. Spectra Data

Compound **1** (21 mg): 13,27-cycloursane, isolated as white amorphous powder, m.p. 151–152 °C; ESI-MS: [M]^+^ at *m*/*z* 410.0, consistent with the molecular formula C_30_H_50_ [M − C_23_H_29_]^+^ at *m*/*z* 105.3; ^1^H-NMR (300 MHz, CDCl_3_) δ ppm: 0.75 (3H, *s*, H-28), 0.89 (3H, *d*, *J* = 6.0 Hz, H-30), 0.98 (3H, *s*, H-26), 1.03 (3H, *d*, *J* = 3.0 Hz, H-29), 1.07 (3H, *s*, H-25), 1.20 (3H, *s*, H-24), 1.28 (3H, *s*, H-23); ^13^C-NMR (75 MHz, CDCl_3_) δ ppm: 39.27 (C-1), 22.30 (C-2), 29.71 (C-3), 30.02 (C-4), 59.51 (C-5), 18.26 (C-6), 41.32 (C-7), 39.72 (C-8), 58.25 (C-9), 37.47 (C-10), 30.52 (C-11), 41.55 (C-12), 38.32 (C-13), 42.16 (C-14), 36.03 (C-15), 32.46 (C-16), 53.12 (C-17), 28.19 (C-18), 42.82 (C-19), 35.04 (C-20), 35.65 (C-21), 32.80 (C-22), 32.11 (C-23), 31.80 (C-24), 20.27 (C-25), 17.96 (C-26), 35.36 (C-27), 14.68 (C-28), 18.68 (C-29), 6.84 (C-30).

Compound **2** (20 mg): 13,27-cycloursan-3-one (Phyllanthone) isolated as white amorphous powder, m.p. 154–155 °C; ESI-MS: [M]^+^ at *m*/*z* 424.3 consistent with the molecular formula C_30_H_48_O, [M − CH_2_]^+^ at *m*/*z* 410.0, [M − C_23_H_29_]+ at *m*/*z* 105.3; UV (CHCl_3_) λmax: 270.50 nm; IR (KBr) υmax cm^−1^: 2948.3 (SP^3^ C-H), 2836.5 (SP^3^ C-H), 1681.0 (C=O of ketone); ^1^H-NMR (300 MHz, CDCl_3_) δ ppm: 0.75 (3H, *s*, H-28), 0.89 (3H, *d*, *J* = 6.0 Hz, H-30), 0.98 (3H, *s*, H-26), 1.03 (3H, *d*, *J* = 3.0 Hz, H-29), 1.07 (3H, *s*, H-25), 1.20 (3H, *s*, H-24), 1.28 (3H, *s*, H-23); ^13^C-NMR (75 MHz, CDCl_3_) δ ppm: 39.27 (C-1), 22.30 (C-2), 213.18 (C-3), 30.02 (C-4), 59.51 (C-5), 18.26 (C-6), 41.32 (C-7), 39.72 (C-8), 58.25 (C-9), 37.47 (C-10), 30.52 (C-11), 41.55 (C-12), 38.32 (C-13), 42.16 (C-14), 36.03 (C-15), 32.46 (C-16), 53.12 (C-17), 28.19 (C-18), 42.82 (C-19), 35.04 (C-20), 35.65 (C-21), 32.80 (C-22), 32.11 (C-23), 31.80 (C-24), 20.27 (C-25), 17.96 (C-26), 35.36 (C-27), 14.68 (C-28), 18.68 (C-29), 6.84 (C-30).

Compound **3** (28 mg): Globraunone, isolated as white amorphous powder, m.p. 220–222 °C; ESI-MS: [M]^+^ at *m*/*z* 554.2, consistent with the molecular formula C_37_H_62_O_3_, base peak M^+^ at *m/z* 554.2, [M + H]^+^ at *m*/*z* 555.0, [M − CH_3_]^+^ at *m/z* 539.1, [M − C_7_H_13_O_3_]^+^ at *m*/*z* 409.9, [M − C_14_H_25_O_3_] + at *m*/*z* 313.6; UV (CHCl_3_) λmax: 229.00 nm, 282.00 nm; IR (KBr) υmax cm^−1^: 3332.2 (OH, *broad*), 2974.4 (SP^3^ C-H), 1656.8 (C=O, weak), 1381.0–1274.7 (C–O). ^1^H-NMR (300 MHz, CDCl_3_) δ ppm: 0.75 (3H, *s*, H-28), 0.89 (3H, *d*, *J* = 6.0 Hz, H-30), 0.97 (3H, *s*, H-26), 1.02 (3H, *d*, *J* = 3.0 Hz, H-29), 1.07 (3H, *s*, H-25), 1.20 (3H, *d*, *J* = 3.0 Hz, H-7′, of C-24), 1.28 (3H, *s*, H-23); ^13^C-NMR (75 MHz, CDCl_3_) δ ppm: 39.27 (C-1), 22.30 (C-2), 213.19 (C-3), 30.02 (C-4), 59.51 (C-5), 18.26 (C-6), 41.32 (C-7), 39.72 (C-8), 58.25 (C-9), 37.47 (C-10), 30.52 (C-11), 41.55 (C-12), 38.32 (C-13), 42.16 (C-14), 36.03 (C-15), 32.46 (C-16), 53.12 (C-17), 28.19 (C-18), 42.82 (C-19), 35.04 (C-20), 35.65 (C-21), 32.80 (C-22), 32.11 (C-23), 20.27 (C-25), 17.96 (C-26), 35.36 (C-27), 14.68 (C-28), 18.68 (C-29), 6.84 (C-30). C-24 Side Chain: 41.74 (C-1′), 72.77 (C-2′), 30.66 (C-3′), 15.80 (C-4′), 35.57 (C-5′), 35.21 (C-6′), 31.80 (C-7′).

Compound **4** (1.22 g): methyl 3,5-dihydroxy-4-methoxybenzoate, isolated as ash amorphous powder, m.p. 160–161 °C; ESI-MS: *m*/*z* 198.0 [M]^+^ consistent with the molecular formula C_9_H_10_O_5_, loss of methoxy at *m*/*z* 167.3 [M − 31]^+^, *m*/*z* 154.3 [M − 44]^+^, loss of methyl ethanoate at *m*/*z* 135.2 [M − 61]^+^, loss of both methoxy and methyl ethanoate at *m*/*z* 107.1 [M − 91]^+^; UV-Vis (MeOH) λmax: 210 nm, 232 nm, 253.0 nm; ^1^H-NMR: (300 MHz, MeOD) δ ppm: 3.85 (3H, *s*, H-1a), 3.91 (3H, *d*, *J* = 3.0 Hz, H-4a), 4.89 (1H, *s*, H-3, H-5), 7.36 (1H, *s*, H-2, H-6); ^13^C-NMR: (75 MHz, MeOD) δ ppm: 55.26 (C-4-methoxy), 59.72 (C-1-methoxy), 106.80 (C-3, C-5), 125.72 (C-1), 142.41 (C-4), 152.90 (C-2, C-6), 168.05 (C-1-ester).

Compound **5** (26 mg): methyl 3-methyl-4-hydroxybenzoate, isolated as yellow semi-solid; ESI-MS: [M + H]^+^ at *m*/*z* 167.7, consistent with the molecular formula C_9_H_10_O_3_, base peak M^+^ at *m/z* 149.2, [M − 61]^+^ at *m*/*z* 105.2; UV-Vis (MeOH) λmax: 212.50 nm, 242.00 nm, 266.50 nm; IR (KBr) υmax cm^−1^: 3385.0 (Phenolic OH, broad), 2939.0 (C-H stretch), 1715.0 (C=O, strong), 1586.0 (C=C aromatic), 1336.3–1123.8 (C–O stretch, strong); ^1^H-NMR: (300 MHz, MeOD) δ ppm: 2.05 (3H, *s*, Me), 3.90 (3H, *s*, -OCH_3_), 4.88 (1H, *s*, -OH), 6.83 (1H, *d*, ^3^*J* = 9.0 Hz, H-5), 7.21 (1H, *s*, H-2), 7.46 (1H, *d*, *J* = 6.0 Hz, H-6); ^13^C-NMR: (75 MHz, MeOD) δ ppm: 20.35 (C-3, Me), 55.38 (C-1, -OCH_3_), 110.74 (C-2), 114.30 (C-5), 116.27 (C-6), 122.43 (C-1), 144.62 (C-3), 150.09 (C-4), 168.74 (C-1-carbonyl ester).

Compound **6** (29 mg): Guaiacol, isolated as a reddish-brown semisolid, ESI-MS: *m*/*z* 125.1 [M + H]^+^ consistent with the molecular formula C_7_H_8_O_2_; UV-Vis (MeOH) λmax: 214.5 nm, 241.5 nm, 271.0 nm; IR (KBr) υmax cm^−1^: 3339.7 (Phenolic OH, strong), 1638.2 (C=C aromatic, medium), 1094.0 (C–O, weak); ^1^H-NMR: (300 MHz, MeOD) δ ppm: 3.91 (3H, *s*, H-2a), 4.87 (1H, *s*, H-1a), 6.73 (1H, *dd*, ^3^*J* = 9.0 Hz, H-3), 6.98 (1H, *t*, *J* = 3.0 Hz, H-4, H-5), 7.10 (1H, *t*, *J* = 3.0 Hz, H-6); ^13^C-NMR: (75 MHz, MeOD) δ ppm: 55.34 (C-6a), 102.52 (C-4), 108.17 (C-5), 114.83 (C-3), 129.39 (C-6), 144.55 (C-2), 147.65 (C-1).

Compound **7** (20 mg): 4-methyl-4-formaldehyde phenone, isolated as brown semi-solid; ESI-MS: *m*/*z* 136.0 [M] + consistent with the molecular formula C_8_H_8_O_2_; *m*/*z* 119.2 [M − 17]^+^, *m/z* 106.8 [M − 29]^+^, *m*/*z* 104.3 [M − 32]^+^; UV-Vis (MeOH) λmax: 234.5 nm, 254.5 nm, 278.50 nm, 291.5 nm; IR (KBr) υmax cm^−1^: 2926.0 (C-H stretch), 1716.4 (C=O), 1459.3 (C=C aromatic); ^1^H-NMR: (300 MHz, MeOD) δ ppm: 1.81 (3H, *s*, H-1a), 7.21 (1H, *d*, *J* = 3.0 Hz, H-2, H-6), 7.74 (1H, *d*, *J* = 3.0 Hz, H-3, H-5), 8.48 (1H, *s*, H-1b); ^13^C-NMR: (75 MHz, MeOD) δ ppm: 22.29 (C-4b), 54.45 (C-4), 125.56 (C-2, C6), 127.19 (C-3, C-5), 176.60 (C-4a), 199.17 (C-1).

Compound **8** (26 mg): 6-methoxy-2H-inden-5-ol, isolated as yellow semi-solid; ESI-MS: *m*/*z* 162.0 [M]+ consistent with the molecular formula C_10_H_10_O_2_; loss of -OH at *m*/*z* 145.0 [M − 17]+, loss of -OCH_3_ at *m*/*z 1*31.0 [M − 31]+, *m*/*z* 114.3 [M − 48]^+^; UV-Vis (MeOH) λmax: 212 nm, 242 nm, 266 nm; IR (KBr) υmax cm^−1^: 3367.6 (Phenolic OH), 2931.6 (SP^3^ − CH), 1586.0 (C=C aromatic, stretch), 1468.0 (C=C, stretch, 5-member ring), 1336.3 (C–O, of an alcohol), 1213.2 (C–O of an alkoxy); ^1^H-NMR: (300 MHz, MeOD) δ ppm: 1.31 (2H, *brs*, H-1), 3.90 (3H, *s*, H-6), 4.88 (1H, *s*, H-5), 7.08 (1H, *d*, H-4b), 7.21 (1H, *t*, H-2a, H-2b), 7.46 (1H, *dd*, H-4a); ^13^C-NMR: (75 MHz, MeOD) δ ppm: 29.35 (C-1), 55.24 (C-6′), 104.89 (C-2), 110.77 (C-9), 120.56 (C-3), 116.81 (C-8), 116.23 (C-4), 108.91 (C-7), 144.63 (C-6), 147.63 (C-5).

### 2.2. Structure Elucidation

The ^13^C NMR spectra of **1** and **2** revealed C-30 compounds, while that of **3** showed 37 signals. The DEPT-135 experiment of **1** showed twelve methylene carbons, twelve methyl and methine carbons and six quaternary carbons. That of **2** differs from **1** by having eleven methylene carbons (one carbon less) attributed to the ketone substituent at δ_C_ 213.18 ppm. ^1^H NMR spectra of the three compounds showed seven methyl protons at δ_H_ 0.75–1.28 ppm, methylene at δ_H_ 1.50–2.32 ppm and methine at δ_H_ 2.50–3.50 ppm. The methyl signals resonated as five singlets and two doublets, which is typical of an α-amyrin (ursane-type) of triterpene.

The signal at δ_C_ 213.18 ppm on both the spectra of **2** and **3** confirmed the presence of a ketone. While the C=O attachment at the C-3 position was based on the HMBC experiment. Upon consideration of the spectra of **1**–**3** and in comparison with spectra data on similar compounds reported in the literature, they were identified as 13,27-cycloursane (**1**), 13,27-cycloursan-3-one previously identified as phyllanthone (**2**) and hexadecahydro-8-hydroxy-9-(2-hydroxy-6-methylheptyl)-1,2,6a,6b,9,12a-hexamethyl-6bHcyclopropa[q]picen-10(11H,12bH,15H)-one (**3**), named globraunone [15,16,17].

The ^1^H NMR of **4** showed four signals, which were two aromatic methoxy at δ_H_ 3.85 and 3.91 ppm, hydroxy at δ_H_ 4.89 ppm and a singlet at δ_H_ 7.36 ppm indicating an aromatic proton. The ^13^C NMR showed nine signals, indicating a C_9_ compound. The signal at δ_C_ 168.05 ppm indicated a carbonyl ester, two pairs of signals, each at 106.80 for olefinic carbons (C-2/C-6) and 152.90 ppm for phenolic carbons (C-3/C-5), typical of an AABB para-substitution pattern [18]. The IR spectrum further corroborated **4** as a hydroxy benzoate with strong bands at 3367.6 cm^−1^ (OH) and 1586.0 cm^−1^ (R-O-C=O). Based on the available spectra data and in comparison, with literature data, **4** was identified as methyl-3,5-dihydroxy-4-methoxybenzoate, previously isolated from *Sacoglottis gabonensis* stem bark [19].

The ^1^H NMR of **5** showed three aromatic protons at δ_H_ 6.83, 7.21 and 7.46 ppm: a phenolic OH at δ_H_ 4.88 ppm and aromatic methoxy at δ_H_ 3.90 ppm. A de-shielded methyl group at δ_H_ 2.01 ppm confirmed that it is directly attached to an aromatic ring. There were nine carbon signals on the ^13^C NMR spectrum. The most de-shielded signal resonated at δ_C_ 168.74. IR spectrum showed having a broad phenolic OH band at 3385.0 cm^−1^, a strong carbonyl C=O band at 1715.0 cm^−1^, C=C aromatic band at 1586.0 cm^−1^ and C–O stretching band at 1336.3–1123.8 cm^−1^. UV absorption at 267 nm showed the excitation of a benzoate skeleton (tabulated as 268 nm). The spectra data of **5** was compared with that of methyl-4-hydroxybenzoate, a bacterial inhibitor previously reported in the bark of *Tsuga dumosa* [20,21], and it was characterized as methyl-3-methyl-4-hydroxybenzoate.

The ^13^C NMR spectra of **6** showed seven signals. Based on the HSQC experiment, the key functional groups identified include aromatic methoxy at δ_C_ 55.34 and δ_H_ 3.91 ppm, four olefinic protons at δ_H_ 6.73, 6.98, 7.10 and 7.20 ppm, with carbon signals at δ_C_ 114.83, 108.17, 129.39 and 110.97 ppm, respectively. The NMR data of **6** agreed with that of guaiacol reported by Kitanovski et al. [22].

^1^H NMR of **7** showed a singlet signal at δ_H_ 8.48 representing an aldehyde carbonyl, two olefinic protons at 7.21 and 7.74 ppm, typical of an A_2_B_2_ para-substitution pattern and a singlet at 1.82 ppm. The aldehydic (1716.4 cm^−1^) and olefinic (1459.3 cm^−1^) bands were prominent on the IR spectrum, while the UV-Vis spectrum showed λmax of 254.5 nm, indicative of a benzene nucleus. Based on the comparison of its spectra data with literature data, **7** was identified as 4-methyl-4-formaldehyde phenone [18].

Compound **8** was showed ten signals on the ^13^C NMR as a C-10, characterized as δ_C_ 55.24 (methoxy), one methylene carbon at δ_C_ 29.35, four methine carbons at δ_C_ 104.89, 108.91, 110.77 and 116.31 and four quaternary carbons at δ_C_ 116.81, 120.56, 144.63 and 147.63 ppm, based on the DEPT135 experiment. HSQC experiment showed that the carbon signals at δ_C_ 104.89 and 110.77 ppm were directly attached to the proton signal at δ_H_ 7.21, while the carbon signals at δ_C_ 116.81 and 108.91 ppm were directly attached to the protons at δ_H_ 7.46 and 7.08 ppm, respectively, thus, confirming an AABC ring system [23].

Three ursane-type triterpenes (**1**–**3**), three phenolics (**4**–**6**) and two phenolic derivatives (**7** and **8**) were isolated and identified in our study of *G. braunii* living on *Leucena leucocephala* (Fabaceae), its host, which marked the first report of these compounds in the plant and the family Loranthaceae. Previous phytochemical studies on the plant have shown the presence of tannins, phenolics, flavonoids, terpenoids and sterols [8,10], while compounds reportedly isolated include lupeol-type triterpenes (globrauneine A-F, lupeol and lupeol acetate), lactones and flavonoids (quercetin, quercitrin, rutin and avicularin), identified in the *G. braunii* living on *Piliostigma thonningi* (Fabaceae). Perhaps, this new additions to the repository of compounds in *G. braunii* might have occurred because of plant-host specificity, which was reported to play a critical role in the quality and quantity of constituents elicited by Mistletoes as well as its influence on their biological properties [4].

**Figure 1:**
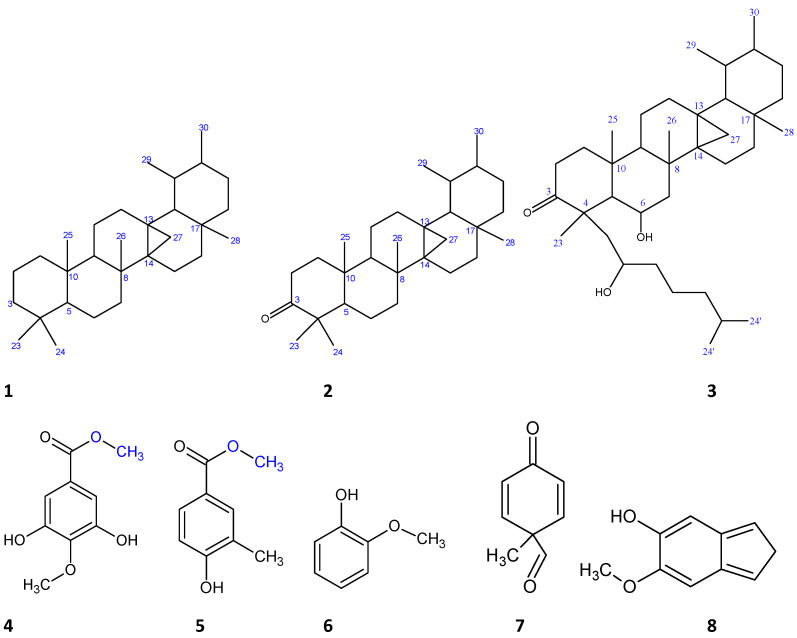
Structures of isolated compounds from the EtOAc fraction of *G. braunii* leaves.

Structures of the isolated compounds **1**–**8** are presented in Figure 1.

### 2.3. Evaluation of the Biological Activities of G. braunii Leaves

**Figure 2:**
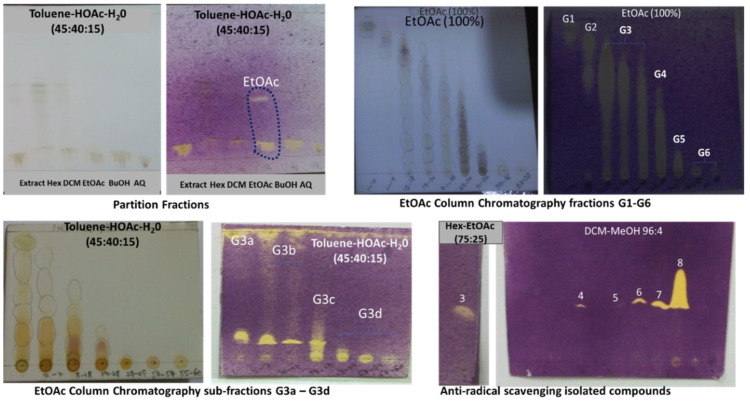
Qualitative antioxidant activity of *G. braunii* leaves by TLC-bioautography against purple DPPH radical solution. **Key:** G1–G6: first column bulk fractions, G3a–G3d: second column bulk fractions, **3**–**8**: isolated compounds.

TLC-bioautography was used throughout the study. This is a reliable and cost-effective technique to isolate lead compounds by employing a suitable chromatographic process, followed by a biological detection system [24]. The TLC-bioautography antioxidant (DPPH) method was reported to demonstrate an interplay of hydrogen atom transfer (HAT) and single electron transfer, which are important mechanisms in understanding the antioxidant properties of natural products [25]. It was used in this study as a guide for rapid and easy identification and isolation of the free radical scavenging compounds present in the leaf extract of *G. braunii*. The result presented in Figure 2 showed that fractionation enhanced the antioxidant property of the plant. The EtOAc fraction **G**, bleached the purple DPPH free radical solution immediately, compared with the *n*-hexane and aqueous fractions, which exhibited a bleaching effect after 5 and 20 min, respectively. The intensity of bleaching (scavenging property) improved with further chromatographic separation.

Quantitative assessments of the antioxidant activity (AOX) of the plant by DPPH, FRAP, TAC and FIC colorimetric assay methods are presented in Table 1. The results showed both concentration-dependent and purification-enhanced increase in the AOX of the plant. The EtOAc fraction exhibited the best AOX among the partition fractions with significant (*p* < 0.05) IC_50_ values of 8.58 and 154.87 µM in the DPPH and FIC assays, respectively.

The former was significantly (*p* < 0.05) better than *L*-ascorbic acid (AA) with an IC_50_ value of 11.38 µM. The power (FRAP) and capacity (TAC) of the EtOAc fraction as an antioxidant were one-sixth and halved, respectively, when compared with AA. This implies that the fraction was able to perform its antioxidant role by hydrogen atom transfer (HAT) to the stable DPPH free radical, single electron transfer (SET) in the FRAP and TAC assays and by metal (Fe^2+^) chelation as in the case of FIC assay [25,26]. These findings corroborate the reported in vivo antioxidant activity of the EtOAc fraction in mice [10].

As presented in Table 1, compounds **1**–**3** had low AOX, while **4**–**8** were active. The ketone group at C-3 position in phyllanthone **2** might have helped to confer one-third fold FRAP activity, while globraunone **3** proved to be the most active among the terpenes isolated partly due to the presence of ketone and hydroxyl groups. This finding is in consonance with that of Baccouri and Rajhi [27] on the significance of hydroxyl, carbonyl and olefine to the antioxidant activities of compounds as lead molecules for drug discovery. Guaiacol **6** demonstrated the best AOX among the isolated compounds. Its AOX was 12 times better as a hydrogen-atom-donor (HAT) than AA in the DPPH assay, while, in the FRAP assay, it was 0.76-times as good as a single-electron-donor (SET) when compared with AA.

This could be due to its *pi* electron-rich benzene ring and hydroxyl group, which can exhibit both HAT and SET mechanisms of antioxidant action. HAT is a one-step reaction by phenolic O-H to effect bond dissociation enthalpy (BDE). SET is a sequential two-step reaction, which entails proton-loss followed by electron-transfer (SPLET), thus, leading to proton affinity (PA) and electron transfer enthalpy (ETE). These mechanisms have been reported to be responsible for the high antioxidant activities of many simple phenolics including phenolic acids [28].

The micro-broth dilution method of microbial susceptibility testing was adopted in the study. It is an objective, high-throughput, cost-effective and quantitative method. It offers high reproducibility, fast generation of MICs, convenience of having pre-prepared panels and the economy of reagents and space that occurs due to the miniaturization of the test, which all make it suitable for the antimicrobial analysis of plant samples. It is also able to assist in generating computerized reports if an automated panel reader is used [29,30].

The antimicrobial activity of the *G. braunii* as shown in Table 2 showed the EtOAc fraction with the best activity among the fraction, based on its lowest MIC range (0.63–5.00 mg/mL) and broad-spectrum activity against the test organisms, which justified our focus on the EtOAc fraction. The tested microorganisms were not strongly susceptible to the terpenes (**1**–**3**) isolated. Globraunone (**3**) was only inhibitory against *B. subtilis* at 2.50 mg/mL and fairly against *C. albicans* at 5.00 mg/mL. This could be due to the ursanoid (α-amyrin), carbonyl and hydroxyl moieties, all playing key roles in the inhibitory action **3** against *C. albicans*. Similar natural compounds, such as ursolic acid and its derivatives, have been reported to exhibit inhibitory actions against *B. subtilis*, MRSA, *P. aeruginosa* and *C. albicans* at 0.10–0.25 mg/mL [31].

On the other hand, the phenolic compounds (**4**–**6**) and their derivatives (**7** and **8**) demonstrated remarkable antimicrobial properties. Guaiacol **6,** a methoxyphenolexhibited the strongest inhibitory activities against *B. subtilis* (0.63 mg/mL) and *C. albicans* (1.25 mg/mL) among the compounds. This was closely followed by dihydroxy-4-methoxybenzoate (**4**) with MIC range of 2.50 and 5.00 mg/mL. However, ciprofloxacin and ketoconazole were 2.5- and 12.5- times better antimicrobial agents compared with guaiacol. Based on the observed antimicrobial activities of the isolated compounds, ranking can be done as follows: Ketoconazole, Ciprofloxacin > Guaiacol **6** > **4** > **8** > **5** > **7** > Globraunone **3** > Phyllanthone **2** > **1** > 50% Aq. MeOH.

Phenolics and its derivatives are referred to as products of the phenylpropanoid pathway, many of which possess significant biological properties. Generally, phenolics with less complex structures (simple phenolics) have been shown to demonstrate remarkable bactericidal and fungicidal activities. These include catechol, caffeic acid, resveratrol and gallic acid amongst others. They all have the phenolic OH and/or other substituents, such as ester, carboxylic, amine, amide and thiol, which have significant antimicrobial properties [32]. Methyl-4-hydroxybenzoate from the bark of *Tsuga dumosa* is a known bacterial inhibitor [20,21], while bulbiferate A and B from *Microbulbifer* spp. are phenolic esters, structurally like compounds **4** and **5**.

According to a report, bulbiferates inhibited the growth of *E coli* and methicillin-sensitive *Staphylococcus aureus* (MSSA) at 0.20 mg/mL [33]. In the same vein, the antioxidant and antimicrobial potentials of natural methoxyphenols, such as eugenol, capsaicin and vanillin have been reported. They exhibited an IC_50_ range of 0.68–1.38 mg/mL against *S. aureus*, 1.21 mg/mL (capsaicin) against *P. aeruginosa* and 2.70 mg/mL (eugenol) against *E. coli* [34].

These leave more to be desired on the biological potentials of natural methoxyphenols and hydroxybenzoates, especially as antioxidant and antimicrobial agents; thus, guaiacol (*O*-methoxyphenol) (**6**) and methyl 3,5-dihydroxy-4-methoxybenzoate (**4**)**,** which were the considerably active antioxidant and antimicrobial compounds identified in this study, could be candidate leads in this respect.

## 3. Materials and Methods

### 3.1. Plant Material

*Globimetula braunii* was collected during the wet season at the Obafemi Awolowo University (OAU), Ile-Ife Campus, Nigeria (GPS Coordinates: Latitude 7.520767, Longitude 4.530315; DMS Lat 7°31′14.7612″ N). It was found parasitizing *Leucena leucocephala* (Lam) De Wit. (Family Fabaceae), its host tree. It was authenticated at the Ife Herbarium, OAU, Ile-Ife, where a herbarium specimen was deposited with Voucher number IFE 17229.

### 3.2. Plant Extraction and Fractionation

The leaves of *G. braunii* were dried at room temperature (25–27 °C). They were powdered (5.0 kg) and macerated with 25 L of EtOH-H_2_O (4:1) at room temperature for 72 h with frequent agitation. This extraction method was a follow-up process to the previous study on the plant [8]. The filtrate was concentrated to dryness in vacuo on a Heidolph RE 400 Rotary Evaporator set at 45 °C and 100 rpm. The extract (300 g) was suspended in distilled water (300 mL) and successively partitioned with *n*-Hexane (600 mL × 4), EtOAc (600 mL × 7) and *n*-BuOH (300 mL × 2).

### 3.3. Qualitative Antioxidant Screening

#### 2,2-Diphenyl-1-picrylhydrazyl (DPPH) Rapid Radical Scavenging Test

Thin-layer chromatography (TLC) bioautography method was used according to Wang et al. [35]. This involved TLC development of the fractions in the appropriate solvent systems in duplicate. The TLC chromatograms were sprayed with 0.5 mg/mL DPPH in MeOH and 10% sulfuric acid. The bleaching effect of the purple DPPH solution by the spots was indicative of the antioxidant potential of the fractions.

### 3.4. Quantitative Antioxidant Screening

All the chemical reagents, solvents and standards used for antioxidant screening were purchased from Sigma Aldrich (St. Louis, MO, USA).

#### 3.4.1. DPPH Spectrophotometric Assay

The DPPH spectrophotometric assay was carried out according to Xiao et al. [36]. A 1 mL DPPH solution in methanol (0.05 mg/mL) was added to 1 mL samples ((positive controls: *L*-ascorbic acid) and (plant fractions/isolated compounds)) at varying concentrations: 50.00, 25.00, 12.50, 6.25 and 3.13 µg/mL. The experiment was carried out in triplicate. The samples were incubated in the dark room for 30 min after which the absorbance was measured at 517 nm on a CamSpec M 107 Spectrophotometer (Spectronics Camspec Ltd., Leeds, UK), where methanol (negative control) was used as the blank. The percentage inhibition of DPPH by each test sample was calculated thus:

(1) $$\%\;\mathrm{Inhibition}\;\mathrm{of}\;\mathrm{sample}\;=\;\frac{{\mathrm{Abs}}_{\mathrm{control}}\;-\;{\mathrm{Abs}}_{\mathrm{sample}}}{{\mathrm{Abs}}_{\mathrm{control}}}\;\times \;100$$

where Abs_control_ = Absorbance of negative control, Abs_sample_ = Absorbance of test sample. The result was expressed as % inhibition and/or IC_50_.

#### 3.4.2. Ferric Reducing Antioxidant Power (FRAP) Assay

This is based on the reduction of the greenish ferric ion (Fe^3+^) 2,4,6-tri-(2-pyridyl)-1,3,5-triazine (TPTZ) to the bluish ferrous ion (Fe^2+^) by natural antioxidants at 593 nm absorbance measurement. The ferric reducing power of plant extracts were determined as ascorbic acid equivalent (AAE) from the calibration curve of the positive control (*L*-ascorbic acid) at concentrations 1000.00, 500.00, 250.00, 125.00, 62.50 and 31.25 µg/mL in methanol [37].

#### 3.4.3. Total Antioxidant Capacity (TAC) Assay

The TAC assay is based on the reduction of Mo^6+^ to Mo^5+^ by the plant samples and subsequent formation of green phosphate/Mo (V) complex at acidic pH according to Prieto et al. [38]. A 0.3 mL extract was combined with 3 mL of reagent solution (0.6 M sulfuric acid, 28 mM sodium phosphate and 4 mM ammonium molybdate). The absorbance of the reaction mixture was measured at 695 nm. The calibration curve was prepared by mixing ascorbic (1000.00, 500.00, 250.00, 125.00, 62.50 and 31.25 µg/mL) with methanol. Data were expressed as mean ± standard error of mean (SEM). The TAC of each sample was expressed as the number of gram equivalent of ascorbic acid (AAE/g).

#### 3.4.4. Ferrous Ion Chelating (FIC) Ability

FIC assay was carried out according to the method of Singh and Rajini [39]. Solutions of 2 mM FeCl_2_·4H_2_O and 5 mM ferrozine were diluted 20 times. An aliquot (1 mL) of different concentrations of extract was mixed with 1mL FeCl_2_·4H_2_O. After 5 min incubation, the reaction was initiated by the addition of ferrozine (1 mL). The mixture was shaken vigorously, and, after a further 10 min incubation period, the absorbance of the solution was measured spectrophotometrically at 562 nm. The percentage inhibition of ferrozine–Fe^2+^ complex formation was calculated by using the formula:

(2) $$\%\;\mathrm{Chelating}\;\mathrm{ability}\;=\;\frac{{\mathrm{Abs}}_{\mathrm{control}}\;-\;{\mathrm{Abs}}_{\mathrm{sample}}}{{\mathrm{Abs}}_{\mathrm{control}}}\;\times \;100$$

### 3.5. Statistical Analysis

All quantitative antioxidant data were analyzed using a One-way Analysis of Variance (ANOVA), followed by the Bonferroni *post-hoc* test on a GraphPad Prism 9 (GraphPad Software Inc., San Diego, CA, USA).

### 3.6. Antimicrobial Test

#### Micro-Broth Dilution Assay

The assay was carried according to the Clinical and Laboratory Standard Institute [40,41]. Bacteria and fungi used for the antimicrobial screening were obtained from the culture collections of the Microbiology Laboratory of the Department of Pharmaceutics, Obafemi Awolowo University where the experiment was conducted. The bacteria and fungi strains were isolated on a Nutrient broth and Sabouraud Dextrose broth (Merck KGaA, Darmstadt, Germany), respectively. The organisms were identified using their morphological characteristics and standard biochemical tests. The reference strains used were *Escherichia coli* ATCC 25923, *Pseudomonas aeruginosa* ATCC 10145, *Bacillus subtilis* NCTC 8236, methicillin-resistant *Staphylococcus aureus* ATCC 29213 and *Candida albicans* ATCC 24433.

Bacteria were maintained on nutrient broth and fungi on Sabouraud Dextrose broth at 4 °C and sub-cultured regularly. Bacteria were grown for 18 h in Nutrient broth and culture suspensions of 10^8^ cfu/mL (equivalent of 0.50 Mc Farland standard) were applied to the dilutions of the fraction/isolates, positive controls (Ciprofloxacin, Ketoconazole) and negative control 50% aqueous methanol employing a multipoint inoculator. Plates were incubated at 37 °C for 24 h. for bacteria strains and 25 °C for 72 h for fungal strains, after which all plates were observed for growth of the microorganisms. The minimum dilution of fractions completely inhibiting the growth and killing each organism was taken as the MIC and MBC/MFC. The sample with the lowest range of MIC and the widest spectrum of activity against bacteria and fungi was taken as the most active.

### 3.7. Isolation of Compounds

#### Column Chromatography of the EtOAc Fraction

The EtOAc fraction (G, 30.0 g) was adsorbed unto 30 g silica gel (70–230 ASTM mesh, Merck KGaA, Darmstadt, Germany), dry-packed on a 600 g silica gel stationary phase within a 300 cm × 5 cm glass column (L x i.d., Fisher Scientific, Waltham, MA, USA). Mobile phase comprising solvent systems of increasing polarity was introduced as thus: *n*-Hex (100%, 700 mL, de-gas), EtOAc (9:1, 8:2, …, 1:9; 500 mL each), EtOAc (100%; 700 mL), EtOAc-MeOH (95:5, 9:1, 8:2, 1:1; 500 mL) and MeOH (100%; 250 mL). Eluates were collected in 20 mL test tubes (1–303). They were bulked into six sub-fractions G1–G6 based on their TLC profiles (SiO_2_, Hex-EtOAc 75:25, 1:1, EtOAc-MeOH 1:1, UV 254 and 365 nm, 10% H_2_SO_4_ spray).

After a 24 h period, sub-fraction G1 (1.2 g) afforded solid deposits a, b and c, while sub-fraction G2 (3.1 g) gave a solid deposit d. Each deposit was washed with 100 mL of MeOH (100%), affording compounds **1**–**4**, respectively. The most active sub-fraction G3 (3.5 g), based on TLC-bioautography was further purified on a Silica gel column with mobile phase from DCM (100%, 100 mL) to DCM-MeOH (98:2, 96:4, …, 80:20; 100 mL each). The eluates (1–187) were collected in 10 mL test tubes and were subsequently bulked into four sub-fractions, G3a–G3d**,** based on their TLC profiles (SiO_2_, DCM-MeOH 97:3, 85:15, 1:1, UV 254 and 365 nm, 10% H_2_SO_4_ spray). A preparative TLC separation of **G3c**, using DCM-MeOH (96:4) afforded bands i–iv, labelled compounds **5**–**8**.

### 3.8. Characterization of Isolated Compounds

Thin-layer chromatography (TLC) of compounds was performed on aluminum-backed silica gel 60 F254 GF plates (0.25 mm, Merck KGaA, Darmstadt, Germany). Chemical detection of the class of compound isolated was done by spraying the developed TLC plates with chromogenic reagents, such as 5% FeCl_3_ for phenolics and 10% H_2_SO_4_ for terpenes. Melting point ranges of the solid compounds were determined on Gallenkamp MPD350-BM 3.5 electrothermal instrument (Gallenkamp, Kent, UK). The UV-Vis absorption was determined within 200–800 nm on Shimadzu UV-1800 UV/Visible Scanning Spectrophotometer: 115 VAC (Shimadzu Corporation, Nkagyo-Ku, Kyoto, Japan). Infrared spectroscopy was done within the 650–4000 cm^−1^ transmittance on Cary 630 FTIR Spectrometer (Agilent Technologies Inc., Santa Clara, CA, USA). ^1^H, ^13^C and 2D (DEPT135, COSY, HSQC and HMBC) NMR spectra of compounds were recorded as solutions on Bruker AMX-300 Spectrometer (Bruker Corporation, Bremen, Germany), where tetramethylsilane was used as the internal standard.

Signals were recorded in the order of chemical shifts (δ) in *part per million* (ppm) relative to the indicated deuterated solvents (CDCl_3_, MeOD), integral values (number of protons), multiplicity (*s*, singlet; *d*, doublet; *t*, triplet; and *m*, multiplet) and coupling constant (*J*) in hertz (Hz). Electrospray Ionization Mass Spectrometry (ESI-MS) was performed on a Varian 500-MS ion trap Mass Spectrometer (Varian, Inc., Palo Alto, CA, USA) for molecular weight determination, expressed in *mass-to-charge* ratio (*m*/*z*). ESI-MS analysis was performed at 10 μL/min sample infusion flow rate; 2.56 kV capillary voltage; 3.0 V extraction cone; 475 L/h desolvation-gas flow rate; 80 and 100 °C for the source- and desolvation-gas temperatures, respectively; and 5.82 mm Vernier-probe-adjuster position. The spectrometer scan range was 99.5–1500.5 *m*/*z* in the positive mode.

## 4. Conclusions

Our activity-guided study on the leaves of *G. braunii* led to isolation of eight compounds (**1**–**8**) from the most active EtOAc fraction. The compounds were identified based on their spectroscopic data and in comparison with literature reports. They were ursane-type triterpenes (**1**–**3**), phenolics (**4**–**6**) and phenolic derivatives (**7** and **8**), all reported for the first time in the plant and in the family Loranthaceae. Guaiacol (**6**) and methyl 3,5-dihydroxy-4-methoxybenzoate (**4**) were remarkably antioxidant with considerable antimicrobial potentials.

## Figures and Tables

**Table 1:** Antioxidant activities of *G. braunii* leaves.

Sample	DPPH (µM)	FRAP (mgAAE/g)	TAC (mgAAE/g)	FIC (µM)
EtOH extract	31.21 ± 1.11 ^g^	109.30 ± 0.76 ^c^	178.15 ± 3.54 ^i^	281.10 ± 12.09 ^e^
*n*-Hexane	75.89 ± 5.05 ^i^	172.88 ± 1.12 ^d^	<10 ^a^	>450 ^h^
EtOAc	8.58 ± 1.39 ^c,^***	178.64 ± 2.04 ^f^	485.81 ± 50.41 ^k^	154.87 ± 6.54 ^b^
*n*-BuOH	16.06 ± 0.52 ^f^	175.38 ± 0.97 ^e^	283.83 ± 23.01 ^j^	298.79 ± 32.51 ^e^
Aqueous	86.97 ± 24.74 ^j^	49.94 ± 18.23 ^b^	<10 ^a^	>450 ^h^
**1**	>100 ^k^	<10 ^a^	12.02 ± 1.37 ^b^	>450 ^h^
**2**	>100 ^k^	347.26 ± 1.43 ^g^	43.91 ± 1.20 ^c^	>450 ^h^
**3**	61.53 ± 1.01 ^h^	651.77 ± 7.98 ^h^	77.72 ± 0.39 ^d^	≥450 ^h^
**4**	6.38 ± 0.48 ^b,^***	702.89 ± 3.09 ^i^	115.23 ± 4.12 ^f^	410.64 ± 8.62 ^g^
**5**	15.78 ± 0.41 ^e^	752.76 ± 13.51 ^k^	121.87 ± 2.73 ^g^	389.92 ± 4.76 ^f^
**6**	0.86 ± 0.37 ^a,^***	720.47 ± 10.08 ^j^	161.57 ± 3.79 ^h^	199.63 ± 5.67 ^c^
**7**	>100 ^k^	764.09 ± 10.12 ^k^	81.12 ± 2.22 ^e^	>450 ^h^
**8**	70.64 ± 2.90 ^i^	634.84 ± 20.31 ^h^	115.76 ± 3.65 ^f^	255.53 ± 11.71 ^d^
Positive control	11.38 ± 0.45 ^d^	-	-	13.21 ± 2.56 ^a^

*n* = 3, values presented as the mean ± SEM, *** significant (*p <* 0.0001) compared with positive control. Values with different alphabets in superscript are significantly different at *p* < 0.05; isolated compounds (**1**–**8**).

**Table 2:** Antimicrobial activities of *G. braunii* leaves.

Sample	Concentration (mg/mL)									
	*E. coli*		*P. aeruginosa*		*MRSA*		*B. subtilis*		*C. albicans*	
	MIC	MBC	MIC	MBC	MIC	MBC	MIC	MBC	MIC	MFC
Negative control	-	-	-	-	-	-	-	-	-	-
EtOH extract	20.00	-	20.00	-	5.00	20.00	20.00	-	10.00	20.00
*n*-Hexane	-	-	-	-	20.00	-	-	-	-	-
EtOAc	2.50	5.00	5.00	10.00	1.25	2.50	0.63	2.50	1.25	2.50
*n*-BuOH	5.00	20.00	2.50	10.00	2.50	5.00	5.00	20.00	1.25	2.50
Aqueous	-	-	-	-	-	-	10.00	-	20.00	-
**1**	-	-	-	-	-	-	20.00	-	-	-
**2**	20.00	-	-	-	-	-	20.00	-	-	-
**3**	10.00	20.00	20.00	-	20.00	-	2.50	10.00	5.00	20.00
**4**	2.50	5.00	5.00	20.00	10.00	-	1.25	5.00	2.50	10.00
**5**	5.00	10.00	10.00	-	20.00	-	2.50	10.00	5.00	20.00
**6**	1.25	5.00	2.50	5.00	2.50	5.00	0.63	1.25	1.25	5.00
**7**	10.00	-	20.00	-	20.00	-	10.00	-	20.00	-
**8**	2.50	5.00	5.00	20.00	2.50	5.00	2.50	5.00	5.00	10.00
Positive control		0.25		0.50		0.50		0.25		0.10

MIC—Minimum Inhibitory Concentration, MBC—Minimum Bactericidal Concentration, MFC—Minimum Fungicidal Concentration; Positive control—Ciprofloxacin (MBC) & Ketoconazole (MFC); isolated compounds (**1**–**8**); negative control = 50% Aq. MeOH; No activity at >20 mg/mL (-).

## Data Availability

Not available.

## Samples Availability

Samples of the compounds **1**–**8** are available from the authors.
